# Safe Crestal Sinus Elevation Below 3 mm Residual Bone with Tissue-Level Implant Placement: A Case Report

**DOI:** 10.3390/reports8040228

**Published:** 2025-11-07

**Authors:** Carola Di Frischia, Marco Tallarico, Marco Gargari, Edoardo Magnifico, Francesco Cecchetti, Francesco Mattia Ceruso

**Affiliations:** 1 Independent Researcher, 00146 Rome, Italy; carola.df.98@gmail.com; 2 Department of Medicine, Surgery and Pharmacy, University of Sassari, 07100 Sassari, Italy; mtallarico@uniss.it; 3 Department of Clinical Sciences and Translational Medicine, University of Rome Tor Vergata, Via Montpellier, 00133 Rome, Italy; marco.gargari@gmail.com; 4 Independent Researcher, 00168 Rome, Italy; edoardomagnifico97@gmail.com; 5 National Institute for Health, 00161 Rome, Italy; cecchetti800@gmail.com; 6 Department of Dentistry “Fra G. B. Orsenigo-Ospedale San Pietro F.B.F.”, 00189 Rome, Italy

**Keywords:** sinus lift, CAS kit, hydraulic sinus lift, bone regeneration

## Abstract

**Background and Clinical Significance**: Maxillary sinus augmentation is a well-established surgical procedure for dental implant placement in the posterior maxilla when the residual alveolar bone height is insufficient. Traditionally, the lateral approach has been preferred in cases with less than 4 mm of bone; however, the crestal approach has emerged as a less invasive alternative, particularly with the advent of advanced techniques and tools such as hydraulic pressure systems and dedicated osteotomy kits. **Case Presentation:** This case report presents the clinical management of a 68-year-old female patient requiring rehabilitation of the right maxillary molars, where the residual bone height measured only 3.6 mm (in position 1.6) and 2.5 mm (in position 1.7). Using the CAS kit system with rounded drills and hydraulic pressure, a controlled crestal sinus elevation was performed, followed by simultaneous implant placement. Despite the extremely limited bone height, a final insertion torque of 30 Ncm was achieved for both implants, likely due to favorable sinus floor anatomy, under-preparation of the implant sites, and the use of tapered, macro-textured implants. Postoperative follow-up over three years showed stable bone levels and successful prosthetic rehabilitation with single crowns. **Conclusions:** This case report highlights the potential of the crestal approach in anatomically challenging scenarios. Proper planning, technique, and implant selection are mandatory to achieve predictable and long-lasting outcomes, even in cases previously considered contraindicated for this method. Further randomized controlled trials are needed to confirm this preliminary result.

## 1. Introduction and Clinical Significance

Maxillary sinus elevation is a common dental procedure performed when there is insufficient alveolar bone height in the maxillary premolar and molar regions. During this procedure, the sinus membrane is carefully separated from the bone surface, creating a space between the membrane and the maxilla. The primary goal of this technique is to increase the height—and eventually the width—of the alveolar bone to allow for dental implant placement.

There are two main techniques for sinus elevation: the lateral and the crestal approach. The first, originally introduced by Tatum in 1975 [1], involves creating an opening in the lateral wall of the maxillary bone to access the Schneiderian membrane. Graft material is placed beneath the membrane to maintain its elevation and promote new bone formation. This technique is generally recommended for patients with less than 4 mm of available bone height [2,3,4]. Depending on the primary stability achievable, implants may be placed either simultaneously with sinus augmentation or in a staged approach after a healing period of 6 to 9 months [5]. In contrast, Summers introduced the crestal approach in 1994 [6], using osteotomes to progressively elevate the sinus floor, thereby reducing surgical trauma compared to the traditional lateral window method. This technique involves accessing the sinus through the alveolar ridge at the intended implant site. A small opening is created in the crestal bone, and specialized instruments are used to gently apply controlled vertical pressure, fracturing the sinus floor while preserving membrane integrity. The space created is filled with bone graft material, and implants are placed simultaneously to enhance the tenting effect and maintain membrane elevation [7]. This technique was initially indicated for cases with a residual bone height of 5–8 mm [8,9]. Known for its minimally invasive nature, the crestal approach appeals to patients seeking reduced surgical trauma and postoperative complications. Over time, modifications have focused on enhancing safety, precision, and predictability, reducing recovery times, and improving long-term implant success rates. Among these advancements is the use of hydraulic pressure to elevate the Schneiderian membrane while simultaneously placing graft material and implants. This has become a gold standard for minimizing membrane perforation by enabling a more controlled and atraumatic lift [10,11,12].

In recent years, the crestal approach has evolved through the introduction of dedicated tools and improvements in implant macro- and micro-design. Rounded drills, drill stops, and advanced implant surfaces have allowed clinicians to treat patients with more severe bone loss, thereby reducing the minimum bone height required for implant placement. Currently, a minimum of 4 mm of residual bone is generally recommended [12]. However, in certain anatomical conditions, the crestal approach may be feasible even with less than 3 mm of bone [13]. According to a recent systematic review, there is no statistically significant difference in the survival rate for implants placed using lateral or transcrestal sinus lift approach procedures, with overall implant survival ranging from 96.9% to 98.9% [14].

This case report aims to assess the feasibility and effectiveness of crestal sinus augmentation in a patient with less than 3 mm of residual alveolar bone height. The objective is to evaluate whether this approach can achieve sufficient bone gain to support implant placement while maintaining the advantages of the transcrestal approach.

## 2. Case Presentation

**Figure 1:**
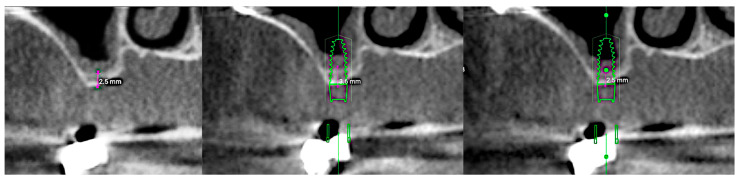
From **left** to **right**: preclinical situation (CBCT), implant planning at the first molar site (residual bone height: 3.6 mm), implant planning at the second molar site (RBH 2.5 mm).

A healthy, non-smoking female patient (68 years old) presented at San Pietro Hospital Fatebenefratelli in Rome in April 2022 for the rehabilitation of two missing maxillary molars (teeth 1.6 and 1.7). Preoperative panoramic radiography (OPT) and cone-beam computed tomography (CBCT) revealed a residual bone height of 3.6 mm and 2.5 mm, respectively, at positions 1.6 and 1.7. There were no signs of sinus pathology or maxillary ostium obstruction (Figure 1).

After discussing the available treatment options with the patient, including a removable prosthesis and a fixed implant-supported restoration with a simultaneous transcrestal sinus lift procedure, the patient elected to proceed with the fixed restoration and declined any removable treatment options. The patient was fully informed about the proposed treatment protocol, and written informed consent was obtained. Data collection ensured complete patient anonymity. As this report documents clinical care without a prospective research component, formal ethical approval was not required in accordance with European and international ethical standards, as well as in accordance with the Declaration of Helsinki (amended in October 2024) (Directive 2001/20/EC) [15]. Institutional Review Board approval was also unnecessary, as no experimental technique was tested, in accordance with applicable legislation (Artt. 10 and 320 cod. civ., and Artt. 96 and 97 of Law No. 633, 22 April 1941). All surgical and prosthetic procedures were performed by an experienced clinician (Francesco Mattia Ceruso).

Prior to surgery, the patient was subjected to professional oral hygiene therapy, including motivational reinforcement, achieving a bleeding-on-probing and full-mouth plaque index of ≤25%. Following local anesthesia, a full-thickness mucoperiosteal flap was elevated to expose the alveolar crest. Osteotomy preparation was carried out using the CAS kit system (Osstem Implant Co., Ltd., Seoul, Republic of Korea), strictly following the manufacturer’s protocol.

- The same step-by-step procedure was performed at both implant sites (1.6 and 1.7):
- Cortical bone marking was performed using a guide drill (2.0/2.7 mm) with a 1 mm stopper, operated at 1000–1500 rpm.
- A 2.2 mm twist drill was used at site 1.6 with a 2 mm stopper (1 mm shorter than the 3.6 mm bone height) at the same speed.
- A 2.8 mm CAS drill, with rounded apical geometry to minimize the risk of Schneiderian membrane perforation, was used with a 3 mm stopper at 400–800 rpm [13].
- A sinus probe with a 4 mm stopper confirmed bone presence at the base of the osteotomy. The same stopper was then used with the 2.8 mm drill to extend preparation by 1 mm.
- A probe with a 5 mm stopper confirmed sinus floor preparation and membrane integrity. A Valsalva maneuver also confirmed the absence of perforation.
- A 3.1 mm CAS drill was then used with the final stopper to slightly widen the implant site, creating under-preparation for better implant stability.
- Hydraulic membrane elevation was performed using sterile saline, infused gradually in 0.5 cc increments to a total of 2.0 cc across both sites.
- At site 1.7, CAS drills (2.8 mm and 3.1 mm) with 3 mm stoppers were used, and an additional 1.0 cc of saline was infused for complete sinus membrane detachment. The first implant site was sealed to prevent saline leakage.

**Figure 2:**
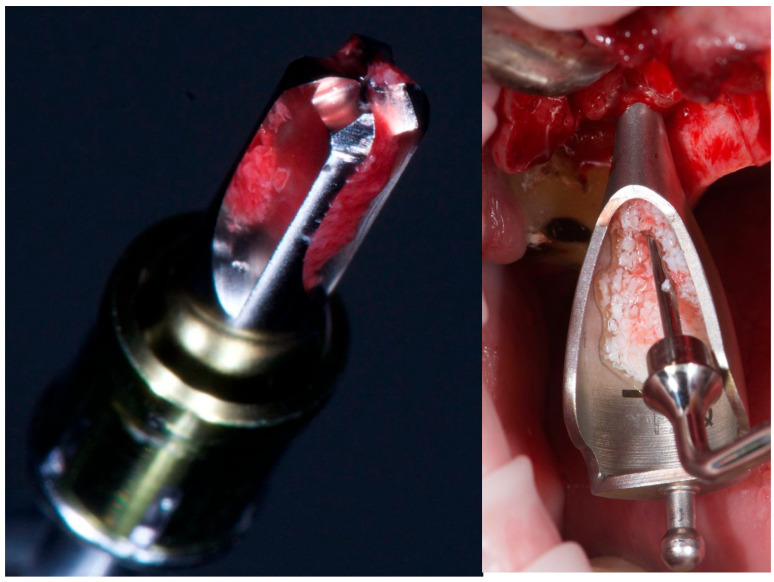
From **left** to **right**: CAS drill showing bone collection, and inorganic bovine bone grafting.

Following sinus elevation, 1 cc of inorganic bovine bone graft (Bio-Oss®, 0.25–1 mm granules, Geistlich Pharma AG, Wolhusen, Switzerland) was inserted—0.5 cc per site (Figure 2).

Two tissue-level, self-tapping implants (PRAMA, Sweden & Martina, Due Carrare, Italy), 3.8 mm in diameter and 10 mm in length, were placed with a final insertion torque of approximately 30 Ncm. These implants feature a 2.8 mm convergent collar with a microtextured UTM surface and a Zirconium–Titanium (ZirTi) body, optimized for the Biologically Oriented Preparation Technique (BOPT). Healing abutments were connected immediately, and the flap was sutured with tension-free closure.

Postoperative instructions were provided, including oral and written guidance, Ibuprofen 600 mg as needed (up to 3 times daily), and Amoxicillin 1 g twice daily for six days. Sutures were removed after one week.

Three months later, a conventional impression was taken. After four weeks, screw-retained porcelain-fused-to-metal crowns were delivered. The patient was enrolled in a hygiene maintenance program, with follow-up extending to three years post-surgery.

**Figure 3:**
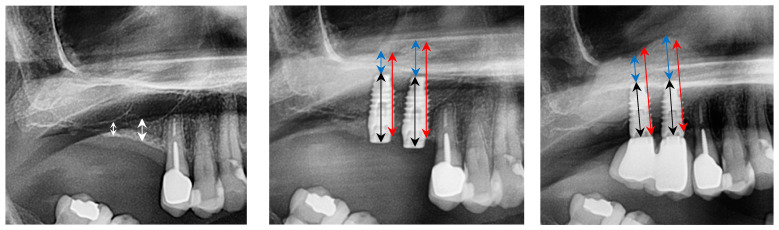
From **left** to **right**: preclinical situation, implant placement, and sinus lift, three years after implant placement. White line: residual bone height (RBH); black line: implant length; red line: overall amount of bone (OAB); blue line: bone above the implant tip (BAI).

No clinical or radiographic complications occurred during the follow-up period. Postoperative bone height increased to 17.8 mm at site 1.6 and 13.8 mm at site 1.7, as measured on panoramic radiographs. This corresponds to a bone gain of 14.1 mm and 11.4 mm, respectively. At the three-year follow-up, bone height remained stable (Figure 3).

## 3. Discussion

Maxillary sinus augmentation is a critical and technically complex procedure when the residual alveolar bone height in the posterior maxilla is less than 4 mm, as such limited bone volume does not provide sufficient support for stable implant placement. This study encourages the crestal approach to sinus elevation, even in patients with less than 4 mm of residual bone height. The postoperative radiograph and the three-year follow-up showed a significant increase in vertical bone height that remained stable over time.

The lateral sinus lift technique is typically indicated when the residual alveolar bone height is less than 5 mm or when a substantial volume of bone graft is required. This approach enables considerable vertical bone augmentation. In contrast, the crestal approach is regarded as less invasive, potentially offering shorter surgical duration and reduced postoperative discomfort. Simultaneous implant placement may be feasible with this method, provided that adequate primary stability is achieved. To minimize the risk of Schneiderian membrane perforation, particularly in cases involving an oblique sinus floor anatomy, the hydraulic pressure mechanism of the CAS kit enables a controlled and progressive elevation of the sinus membrane. Also, the use of rounded drills and depth-control stoppers further improves safety, reducing the risk of damaging the membrane [13].

In scenarios where simultaneous implant installation is desired and bone conditions permit, the crestal technique may be considered the gold standard approach. Although the crestal approach for sinus augmentation offers several clinical advantages, it is not devoid of potential risks and complications. A primary concern is the possibility of Schneiderian membrane perforation, which may adversely affect graft integration and increase the risk of postoperative sinusitis or infection. The limited visual and tactile control associated with this technique can further elevate the likelihood of membrane damage, especially in cases presenting a thin or anatomically irregular sinus floor. Ultimately, the choice of technique should be guided by the clinician’s expertise, patient-specific anatomical considerations, and individual clinical requirements.

Recent advances have seen the crestal approach applied in more severe cases of bone loss, even when larger volumes of grafting material are necessary. However, this approach presents an increased risk of sinus membrane perforation, especially when residual bone height is critically low. The lateral technique, by providing direct visualization and manipulation of the sinus membrane, offers greater control and thereby minimizes the risk of complications [2,3,4,8].

In sinus augmentation procedures, a variety of bone graft materials can be employed, each offering distinct advantages based on the clinical context. Autogenous bone, harvested from the patient (mandibular ramus or iliac crest), was widely used in the past due to its osteogenic potential, as it contains viable cells that actively contribute to bone regeneration [16,17,18]. In recent years, xenografts—commonly sourced from bovine or porcine origin—have been extensively processed to remove organic components and minimize immunogenicity; they serve primarily as an osteoconductive scaffold [19]. Additionally, synthetic grafts such as hydroxyapatite, β-tricalcium phosphate, and bioactive glasses are engineered to mimic natural bone structure and facilitate new bone formation by acting as biocompatible scaffolds [20,21].

This case presented an additional level of complexity due to the presence of two adjacent implant sites. It was crucial to temporarily seal the first site during saline injection at the second site, as hydraulic pressure naturally follows the path of least resistance. Sealing the first site ensured proper maintenance of pressure within the sinus cavity and allowed for controlled, uniform membrane elevation.

Moreover, even with only 2.5 mm of residual bone at one site, a final insertion torque of approximately 30 Ncm was achieved. This result may be explained by favorable sinus floor anatomy, which provided additional bone at the medial and distal aspects of the implant site. Furthermore, the under-preparation of the implant site and the macro-design of the implant—featuring a tapered body and self-tapping threads—likely contributed to high primary stability. These findings highlight the importance of careful preoperative planning using 3D radiographic imaging, as well as the selection of implant systems that support under-preparation at compromised sites.

From a prosthetic standpoint, the use of single crowns may offer both esthetic and hygienic advantages. Esposito et al. [22] demonstrated positive medium-term outcomes using single crowns on short implants, even in the posterior mandible. This approach facilitates plaque control and maintenance while maintaining satisfactory esthetic outcomes.

The choice between lateral and crestal sinus floor elevation techniques remains clinically significant, particularly in cases with limited residual bone height in the posterior maxilla. Current evidence indicates comparable implant survival rates between the two approaches when proper case selection and surgical protocols are followed [14]. A recent advancement in sinus surgery includes a minimally invasive crestal approach technique with specially designed burs operating in a non-cutting, counterclockwise direction to compact and autograft bone along the osteotomy walls. This process, named osseodensification, could enhance bone density and stability, allowing for controlled elevation of the sinus floor without perforating the Schneiderian membrane [23]. Finally, crestal techniques—especially those employing osseodensification—are associated with lower complication rates, greater primary stability, and reduced postoperative morbidity compared to the lateral window approach. While the lateral technique remains the standard for extensive vertical augmentation, recent studies support the crestal approach as a less invasive and equally predictable alternative in moderate cases [23].

Finally, future developments could focus on the use of implants with hydrophilic surfaces. These surfaces enhance early osseointegration by promoting protein adsorption and osteoblast adhesion, thereby improving early implant stability. The application of such implant surfaces in crestal sinus augmentation procedures—particularly in cases with residual bone height less than 3 mm—could increase the predictability and long-term success of the treatment [24,25].

## 4. Conclusions

The use of the CAS kit system in combination with tissue-level implants resulted in substantial vertical bone gain even in the case of reduced bone height (less than 3 mm). No intraoperative or postoperative complications were observed. Additional studies involving larger sample sizes and long-term follow-up are necessary to further validate these outcomes and assess their reproducibility over time.

## Data Availability

The original contributions presented in this report are included in the article. Further inquiries can be directed to the corresponding author.

## References

[1] Chen L., Cha J.K. (2007). Clinical and radiographic evaluation of sinus floor augmentation using a crestal approach with osteotomes and various grafting materials. Int. J. Oral Maxillofac. Implant..

[2] Esposito M., Grusovin M.G., Rees J., Karasoulos D., Felice P., Alissa R., Worthington H., Coulthard P. (2010). Effectiveness of sinus lift procedures for dental implant rehabilitation: A Cochrane systematic review. Eur. J. Oral Implantol..

[3] Jensen O.T. (1999). The Sinus Bone Graft.

[4] Pjetursson B.E., Tan W.C., Zwahlen M., Lang N.P. (2008). A systematic review of the success of sinus floor elevation and survival of implants inserted in combination with sinus floor elevation. J. Clin. Periodontol..

[5] Del Corso M., Vervelle A., Simonpieri A., Jimbo R., Inchingolo F., Sammartino G., Dohan Ehrenfest D.M. (2012). Current knowledge and perspectives for the use of platelet-rich plasma (PRP) and platelet-rich fibrin (PRF) in oral and maxillofacial surgery. Curr. Pharm. Biotechnol..

[6] Stübinger S., Landes C., Seitz O. (2010). Palatal piezosurgical window osteotomy for maxillary sinus augmentation. Int. J. Oral Maxillofac. Surg..

[7] Summers R.B. (1994). A new concept in maxillary implant surgery: The osteotome technique. Compendium.

[8] Tatum H. (1986). Maxillary and sinus implant reconstructions. Dent. Clin. N. Am..

[9] Wallace S.S., Froum S.J. (2003). Effect of maxillary sinus augmentation on the survival of endosseous dental implants: A systematic review. Ann. Periodontol..

[10] Soltan M., Smiler D.G. (2005). Antral membrane balloon elevation. J. Oral Implantol..

[11] Fugazzotto P.A., Vlassis J. (2003). A Simplified classification and repair system for sinus membrane perforations. J. Periodontol..

[12] Testori T., Wallace S.S., Del Fabbro M., Taschieri S., Trisi P., Capelli M., Weinstein R.L. (2008). Repair of large sinus membrane perforations using stabilized collagen barrier membranes: Surgical techniques with histologic and radiographic evidence of success. Int. J. Periodontics Restor. Dent..

[13] Gatti F., Gatti C., Tallarico M., Tommasato G., Meloni S., Chiapasco M. (2018). Maxillary sinus membrane elevation using a special drilling system and hydraulic pressure: A 2-year prospective cohort study. Int. J. Periodontics Restor. Dent..

[14] Shah D., Chauhan C., Shah R. (2022). Survival rate of dental implant placed using various maxillary sinus floor elevation techniques: A systematic review and meta-analysis. J. Indian Prosthodont. Soc..

[15] European Commission—European Medicines Agency (2007). Report on the Conference on the Operation of the Clinical Trials Directive (Directive 2001/20/EC) and Perspectives for the Future. EMEA/565466/2007. https://health.ec.europa.eu/system/files/2017-02/ec_emea_conference_on_clinical%252520_trials_en_0.pdf.

[16] Aghaloo T.L., Moy P.K. (2007). Which hard tissue augmentation techniques are the most successful in furnishing bony support for implant placement?. Int. J. Oral Maxillofac. Implant..

[17] Nkenke E., Stelzle F. (2009). Clinical outcomes of sinus floor augmentation for implant placement using autogenous bone or bone substitutes: A systematic review. Clin. Oral Implant. Res..

[18] Dellavia C., De Colli M., Sartori S., Tettamanti L. (2014). Use of allogenic bone in sinus augmentation procedures: A review. Oral Implantol..

[19] Artzi Z., Weinreb M., Givol N., Rohrer M.D., Nemcovsky C.E., Prasad H.S., Tal H. (2004). Biomaterial resorption rate and healing site morphology of inorganic bovine bone and beta-tricalcium phosphate in the canine: A 24-month longitudinal histologic study and morphometric analysis. Int. J. Oral Maxillofac. Implant..

[20] Hench L.L. (1991). Bioceramics: From Concept to Clinic. J. Am. Ceram. Soc..

[21] LeGeros R.Z. (2002). Properties of osteoconductive biomaterials: Calcium phosphates. Clin. Orthop. Relat. Res..

[22] Esposito M., Gatti F., Meloni M., Muzzi L., Baldini N., Buti J., Minciarelli A., Xhanari E., Tallarico M. (2023). To splint or not to split short dental implants under the same partial fixed prosthesis: Five-year results from a multicentre randomized controlled trial. Clin. Trials Dent..

[23] Pereira J.F., Costa R., Vasques M.N., Salazar F., Mendes J.M., da Câmara M.I. (2023). Osseodensification: An Alternative to Conventional Osteotomy in Implant Site Preparation: A Systematic Review. J. Clin. Med..

[24] Buser D., Broggini N., Wieland M., Schenk R.K., Denzer A.J., Cochran D.L., Hoffmann B., Lussi A., Steinemann S.G. (2004). Enhanced bone apposition to a chemically modified SLA titanium surface. J. Dent. Res..

[25] Schwarz F., Wieland M., Schwartz Z., Zhao G., Rupp F., Geis-Gerstorfer J., Schedle A., Broggini N., Bornstein M.M., Buser D. (2009). Potential of chemically modified hydrophilic surface characteristics to support tissue integration of titanium dental implants. J. Biomed. Mater. Res. Part B Appl. Biomater..

